# Developing and Assessing the Acceptability of an Information Booklet for Patients in Surveillance for Abdominal Aortic Aneurysms: An Intervention Development Study

**DOI:** 10.1111/hex.70631

**Published:** 2026-03-10

**Authors:** Cheryl Grindell, Jane Hughes, Elizabeth Lumley, Alan Elstone, Jo Hall, Jonathan Michaels, Akhtar Nasim, Stephen Radley, Phil Shackley, Niall MacGregor Smith, Gerry Stansby, Emily Wood, Alicia O'Cathain

**Affiliations:** ^1^ Sheffield Centre for Health and Related Research (SCHARR), Division of Population Health The University of Sheffield Sheffield South Yorkshire UK; ^2^ University Hospitals Plymouth NHS Trust Plymouth Devon UK; ^3^ Derbyshire Community Health Services NHS Foundation Trust Chesterfield Derbyshire UK; ^4^ Sheffield Vascular Institute, Sheffield Teaching Hospitals NHS Foundation Trust, Northern General Hospital Sheffield South Yorkshire UK; ^5^ c/o PCAAAS, The University of Sheffield Sheffield South Yorkshire UK; ^6^ University and Newcastle upon Tyne Hospitals NHS Foundation Trust, Freeman Hospital Newcastle upon Tyne Tyne and Wear UK

**Keywords:** abdominal aortic aneurysm, information booklet, surveillance

## Abstract

**Background:**

In the United Kingdom and Sweden, men aged 65 are offered screening for Abdominal Aortic Aneurysm (AAA). Men with small AAA enter a surveillance programme to monitor growth until the AAA is large enough for referral for treatment. Some men develop anxiety related to having an AAA or being in surveillance.

**Aim:**

The original aim was to develop and assess the acceptability of a new intervention to help men in surveillance manage anxiety. As the study progressed, the aim changed to developing an information booklet to address men's uncertainties about AAA and surveillance because uncertainties might lead to anxiety.

**Method:**

An intervention development study was undertaken, following guidance in three phases: 1. Identifying need using surveys of screening staff and men, and qualitative interviews with men and family members; 2. Co‐design of an intervention using a literature review, programme theory development, and two workshops with men, family members, and a patient representative; 3. Assessing the acceptability of the intervention using a telephone survey of 23 men in AAA surveillance.

**Result:**

Although the original aim was to develop an intervention to help men manage anxiety, men and screening staff identified the need for an information‐based intervention to address men's uncertainties about AAA. Published evidence identified that uncertainty about health conditions or treatment can lead to anxiety, so the intervention taken forward was an information booklet to address men's uncertainties about AAA. A 16‐page A5 booklet was developed with men and their family members, addressing why men had to wait for AAA to become large before referral for treatment, the risk of rupture for different sizes of AAA, and how to reduce the risk of rupture. In the telephone survey, 20/23 men in the AAA surveillance who read the draft booklet found it helpful because it addressed their uncertainties. They suggested minor refinements. A refined version of the prototype booklet was produced based on this feedback.

**Conclusions:**

A co‐designed information booklet is available that addresses the uncertainties of men with AAA in surveillance. Future research should measure the impact of the information booklet on AAA‐related anxiety.

**Patient and Public Contributions:**

We set up a patient panel specifically for this study. We identified five men from different sources including asking the vascular clinicians on the team to invite patients to consider joining the panel and approaching existing health research studies patient panels to identify men who had AAA. One member of our team was the patient representative on the research committee for the NHS AAA Screening Programme in England. He attended all our patient panel meetings. The panel reviewed all the documents we used to invite men to different parts of the study, offered advice about recruitment, and gave feedback about the findings. They offered advice about the prototypes of the information booklet.

**Abbreviations**
AAA: abdominal aortic aneurysm
IMD: index of multiple deprivation
NHS: National Health Service
PPIE: Patient and Public Involvement and Engagement
SD: Standard deviation
UK: United Kingdom

## 1 Introduction

Screening programmes are established to save lives. However, they may also raise anxiety levels and detrimentally affect health‐related quality of life [1]. An Abdominal Aortic Aneurysm (AAA) is an enlargement in the aorta, which is the main artery that carries blood from the heart to the rest of the body [2]. In some countries, people are screened for AAA because AAAs can increase in size over time and may rupture, which can be immediately life‐threatening [1, 2]. People with an AAA between 3.0 and 5.5 cm (small/medium) enter surveillance, where they are monitored until their AAA is large enough for referral for treatment. Sweden and the United Kingdom (UK) have national AAA screening programmes for men [3, 4]. Only men are invited for screening because they are four times more likely to have an AAA than women [5]. In the UK, each nation has a National Health Service (NHS) screening service for men when they reach the age of 65 [6]. Men with a small or medium AAA enter surveillance and are scanned annually or every 3 months, respectively.

The existing evidence about whether AAA diagnosis and surveillance cause psychological distress is mixed. Two Swedish studies, using health questionnaires [7] and qualitative interviews [8], explored men's and their partners' experiences of living with AAA. They identified that some men and family members are anxious when diagnosed with AAA [7, 8]. The most recent systematic review of quantitative studies, which included studies using generic quality of life measures, concluded that current evidence did not support a negative impact on quality of life [9]. Recent quantitative studies of psychological distress from AAA surveillance in multiple countries, using AAA‐specific measures of quality of life, identified that a minority of those in surveillance suffer significant amounts of AAA‐related anxiety [1, 10, 11]. Therefore, there appears to be a need for an intervention to help at least some men in surveillance to manage AAA‐related anxiety.

In recent publications from our team, we identified that doctors, nurses and technicians providing AAA screening in the UK thought that some men were anxious and were supportive of an intervention to address this anxiety [12]. They suggested that organised support groups, improved provision of information, and more interaction with the screening service might help. Our survey of men in surveillance identified that most men reported that they were not anxious, but a minority of men reported being anxious all or most of the time [11]. Our follow‐up qualitative interviews with men in surveillance, who had reported AAA‐related anxiety in the survey, suggested that they did not necessarily consider themselves to be anxious. Instead, men identified multiple uncertainties about AAA and the screening process [13]. To address these uncertainties, they wanted both written information and verbal communication with health professionals, particularly in relation to what they could or should not do to prevent rupture. Many of the men did not consider counselling and talking‐based interventions as helpful [11]. The need for information about AAA, screening and treatment is highlighted in European guidance for AAA [14]. It is also the case that uncertainty is not unique to AAA surveillance. It has also been found to be a key issue for men in active surveillance for prostate cancer [15]. Information and education have been recommended to address the psychosocial burden for men in this context [16] and in surveillance for other cancers [17, 18].

In summary, there is a need for an intervention to help men address AAA‐related anxiety. We established a study to fill this research gap by developing and assessing the feasibility of an intervention to address men's AAA‐related anxiety. However, as our study progressed, the aim narrowed to developing and assessing the acceptability of a written information resource for men in AAA surveillance to address their uncertainties about AAA and being in surveillance. The rationale was that uncertainties about AAA and surveillance can lead to anxiety.

## 2 Methods

### 2.1 Setting

In England, men aged 65 are screened for AAA. They enter surveillance if a small or medium AAA is detected or if their AAA is already large enough, they are referred to a vascular surgeon. NHS screening and surveillance are delivered by 38 regional screening providers. In 2023/24, there were approximately 15,300 males in surveillance in the NHS AAA Screening Programme in England [6].

### 2.2 Design

We undertook an intervention development study. The original aim was to develop an intervention to manage AAA‐related anxiety for men in AAA surveillance. When we started the study, we made the assumption that any intervention to address men's AAA‐related anxiety was likely to be a complex intervention with multiple components. Therefore, we followed the first phase of the MRC framework for the development and evaluation of complex interventions [19]. Details of how to develop complex interventions are documented in MRC‐funded guidance [20]. This intervention development guidance offers a set of actions to consider. We addressed these actions in three phases: identifying the need for an intervention, co‐design of the intervention, and assessing the acceptability of the prototype intervention. Phase 1 (need for the intervention) has been reported in three publications [11, 12, 13], and the findings are summarised in this current paper. Phases 2 (co‐design) and 3 (acceptability) are reported fully in this paper. The relationship between each intervention development action from the guidance, and each method used within this study, is summarised in Table 1.

**TABLE 1:** Relationship between intervention development guidance and methods used.

	Phase 1 needs assessment			Phase 2 co‐design			Phase 3 acceptability
Actions	Staff survey	Patient survey	Interviews with patients and family members	Literature search	Theory and programme theory	Co‐design workshops	Acceptability telephone survey
Involving stakeholders	x	x	x	—	—	x	x
Reviewing published research evidence	—	—	—	x	—	—	—
Drawing on existing theories and articulating programme theory	—	—	—	—	x	—	—
Undertaking primary data collection	x	x	x	—	—	—	x
Understanding context	x	x	x	—	—	x	—
Paying attention to future implementation in the real world	x	—	x	—	—	x	x
Designing and refining an intervention using iterative cycles of development with stakeholder input throughout	—	—	—	—	—	x	x

*Note:* (x) indicates which method was undertaken for each action, (—) not undertaken.

This paper follows the GUIDED checklist for reporting intervention development studies in health care [21].

### 2.3 Ethics

We obtained ethics approval from the Wales REC6 Ethics Committee, IRAS project ID 321528. We obtained written informed consent from all participants.

### 2.4 Phase 1: Identifying the Need for an Intervention

We undertook an online survey of service providers [12], a postal survey of men in surveillance [11], and a qualitative interview study of men who reported AAA‐related anxiety in the survey [13]. The methods of phase 1 are reported elsewhere.

### 2.5 Phase 2: Co‐Design of an Intervention

Based on the needs assessment, it was obvious that one component of any intervention would need to address the information deficit felt by some men and service providers. At this point, our aim changed from developing an intervention to help men in AAA surveillance manage anxiety, to developing a booklet to address men's information uncertainties about AAA. Based on the Phase 1 findings, we set out to co‐design the content and layout of an information booklet using the following seven steps.

#### 2.5.1 Literature Review

In August 2024, we searched for existing information sources for patients with AAA. At this time, we found the NHS leaflet given to all men who are screened in England, two leaflets available on the NHS website for people diagnosed with small or medium aneurysms, and a decision aid for men with large AAA facing the decision about the type of treatment to have. Parts of these leaflets addressed some of the knowledge gaps identified in our primary research [13]. We also carried out a scan of the literature on the effectiveness of patient information materials using the ‘pearl growing’ search technique [22, 23]. Our review of the literature was intended to identify key issues to consider when developing this information booklet rather than be a systematic or exhaustive search of available research (see Appendix 1 for a description of the search). Reviews were prioritised where available. This resulted in 27 articles published in the last 10 years. We used these articles to provide a summary of what is known about patient information in AAA or similar conditions, the communication of risk, and the development of patient information materials. These sources were used to facilitate the development of the information booklet.

#### 2.5.2 Production of Version 1 of Prototype Booklet

We produced an outline of the different sections that could be included in the booklet. The Patient and Public Involvement and Engagement (PPIE) group discussed this outline and two existing AAA leaflets, which included some of the information relevant to the planned booklet. Then a prototype of the booklet was produced and refined through discussion with team members. Team members included two vascular surgeons, a retired vascular surgeon, the Clinical Lead for the AAA screening programme in England, the nurse lead for the AAA screening programme, and a man who had had AAA. Two AAA Specialist Nurses also commented on the prototype.

#### 2.5.3 Co‐Design Workshop 1

We undertook workshops as a research method [24]. The workshop activities attended to the co‐design principles of bringing people together as equal partners, where all experiences and ideas would be valued [25, 26, 27]. It was a 4 h in‐person workshop with four men in AAA surveillance, one family member, an AAA specialist nurse, one PPIE member, a clinical psychologist, and two academics. Men and their family members gave written informed consent for participation. We recruited men who lived within travel distance of the venue in the north of England and who had agreed to further contact when responding to our patient survey [11]. We sent 58 invitations, and when men agreed to participate, we sent them the draft booklet before the meeting. The workshop was facilitated by two academics. In the workshop, findings from phase 1 of our intervention development process were described, including the proposed solutions generated from our surveys and interviews. We asked men what interventions they felt we should take forward, discussed the draft booklet, including the content and how best to use any booklet, and asked if there were other parts of an intervention that were needed.

#### 2.5.4 Theory and Programme Theory

If we had stayed with our original aim, we would have considered theories about anxiety management and based our intervention on these. Because our aim changed to developing an information booklet to address men's uncertainties, we instead developed a logic model to capture the programme theory of how this information intervention might operate to affect short‐term, intermediate and long‐term outcomes for men in surveillance. In addition, we searched for theories of patient health information seeking and considered their relevance to the intervention.

#### 2.5.5 Production of Version 2 of the Prototype Booklet

Based on feedback from workshop 1, we expanded the prototype booklet and redesigned parts of it. More details regarding these changes can be found in later parts of the results section. We then worked with team members and our PPIE panel to produce the next version.

#### 2.5.6 Co‐Design Workshop 2

We brought together the workshop 1 participants in a 90 min online workshop. Four men and one family member attended. We sent them the latest version of the booklet before the meeting, along with a list of changes we had made based on workshop 1. The workshop was facilitated by an academic. We summarised feedback from the first workshop, presented the logic model, discussed ways of improving the booklet, and discussed how best to implement it.

#### 2.5.7 Finalising the Prototype Booklet for Phase 3

We refined the prototype based on workshop 2 feedback. The product was an A5 booklet on high‐quality paper, 16 pages long, with diagrams and illustrations to support the text.

### 2.6 Phase 3: Acceptability of the Prototype Booklet

We conducted a telephone survey to review the draft booklet. Survey methodology was used because the aim was to measure the acceptability of the booklet. The survey was undertaken by telephone rather than post to obtain detailed feedback on the utility of the booklet and how it could be improved. The aim was to recruit 20 men who were currently in the AAA surveillance programme. Sampling was done incrementally to achieve a spread of participants from both affluent and socially deprived areas using the Index of Multiple Deprivation quintiles [28]. See Appendix 2 for the sampling approach.

Once a participant responded to the invitation, we arranged a telephone call to complete a researcher‐administered survey exploring opinions of the booklet, whether they perceived they had gained any benefit from reading it, and any improvements they would like to see. Part of the survey included the QQ‐10, a validated instrument designed for obtaining views of patient‐reported outcome measures [29]. The survey also comprised open questions to enable more detailed responses (see Appendix 3 for the survey). The researcher made written notes during the telephone call. Quantitative data were entered into SPSS [30], and descriptive statistics were used to identify the percentage of men finding the booklet acceptable. The items in the QQ‐10 equate to two domains of ‘value’ of the booklet and ‘burden’ of the booklet. The aim is to achieve a high score for ‘value’ and a low score for ‘burden’. We analysed the notes from the open questions by reading and coding them into themes [31].

Based on the findings of the telephone survey, we refined the booklet further.

## 3 Results

### 3.1 Phase 1: Identifying the Need for an Intervention

We summarised key findings from the two surveys and qualitative interview study against a set of questions around whether an intervention was needed and what it might look like (see Table 2). Prior to this needs assessment, we had identified potential interventions as cognitive behavioural therapy for men with high levels of anxiety, peer support programmes, and digital health interventions to provide scalable solutions to address anxiety. However, it was clear from the phase 1 research that there was an information gap that needed to be filled to address men's uncertainties about AAA and AAA surveillance because these uncertainties might contribute to anxiety.

**TABLE 2:** Key findings from the needs assessment.

Question	Staff survey [10]	Patient survey [9]	Qualitative interviews with men reporting anxiety on the survey and their family members [11]	Conclusion
Are men anxious?	Yes	The majority of 734 men reported not being anxious. 11% (29/257) of men in three monthly surveillance reported being anxious most or all of the time.	Some men said they were anxious but most said not, even though they reported anxiety in the survey. They described anxiety at the time of diagnosis and when their next scan was due. They said they ‘thought about’ the AAA rather than feeling anxious	An intervention could target men reporting anxiety but given the challenges around whether men will open up about having anxiety, it may be best to offer the intervention to all men in surveillance.
Is an intervention needed?	85% (72/84) of staff agreed men in surveillance may need help to manage anxiety	Not asked in the closed questions One of our findings from the open question was men saying they wanted more information provision	Men had many uncertainties about AAA screening, so they wanted information to address these	An intervention is needed to address uncertainties about AAA and AAA surveillance, which may contribute to anxiety
What options are there for interventions?	Improved information about AAA and risks Group support/networking sessions More contact with screening service staff. Programme nurses are best to deliver interventions	In open comments 11% (28/246) commented about a lack of information about AAA and screening	Most men did not want talking therapy‐based interventions. They wanted information provided by health professionals and written information They wanted information tailored to their situation that was trustworthy They wanted further contact with the AAA screening programme	Written information would be valued. Further contact with an AAA nurse would be valued too but this required a resource commitment by the NHS AAA screening programme
Who should receive the intervention?	63% (51/80) said it should be offered to all men, rather than targeted at men specifically identified as anxious	Men who reported anxiety were more likely to be younger, from socially deprived communities, have poorer physical health, have larger and faster‐growing AAAs.	Not addressed	Offer an intervention to all men in surveillance.
What is the required content of any information?	Risk of rupture Future treatment	Why wait for surgery? Symptoms to look out for Do's and don'ts Hereditary nature of AAA Future treatment	What to do, and not do, to prevent rupture How AAA is measured Decision‐making about having surgery and options available	Written information should include the concerns raised within different parts of the needs assessment
Format/mode of delivery/aspects of intervention	Available in different languages 66% (54/81), and offered face‐to‐face 33% (27/80). Most highly rated as important (49% (39/79)) was the intervention having different parts so men can pick and choose what they want to use. Telephone helpline Specialist Nurse	Not addressed in closed questions	Information from a trustworthy source Written information, and health professional delivered maybe via follow‐up telephone call Tailored to individuals	Start the process by developing a written information component but it may need to be added to in the future with a meeting or a telephone call with a Specialist Nurse.

### 3.2 Phase 2: Co‐Design of the Intervention

#### 3.2.1 Contribution of the Literature Review

We identified a number of leaflets available to men from the NHS AAA screening service aimed at different stages of the disease and surveillance process. Our literature search identified 27 publications [32, 33, 34, 35, 36, 37, 38, 39, 40, 41, 42, 43, 44, 45, 46, 47, 48, 49, 50, 51, 52, 53, 54, 55, 56, 57, 58] that informed the intervention development. This evidence base showed that although information could improve anxiety, existing AAA information on general websites had a high reading age and was not comprehensive. Also, we would need to attend to reading age, good ways of communicating risk, such as providing information about how smoking increases the risk of AAA growth, and promote gains not losses, for example, the benefits of stopping smoking to slow AAA growth, if we wanted to change behaviour (see Appendix 4 for the learning from the literature).

#### 3.2.2 Contribution of Workshop 1

Workshop 1 participants liked the idea of the booklet and the list of headings presented. They felt that it would be very useful but wanted the following changes: more information about support for stopping smoking, information about medications that may be prescribed, and more detailed information about how the aorta is measured. They also made suggestions about how to make the layout clear, for example, using more colour, larger section headings, and space to make notes. They did not have views on alternatives to the booklet, or other components of the intervention. It was evident that the participants valued the provision of more detailed information because they took the opportunity during the workshops to ask the attending AAA specialist nurse multiple questions.

#### 3.2.3 Contribution of Theory and Programme Theory

**FIGURE 1:**
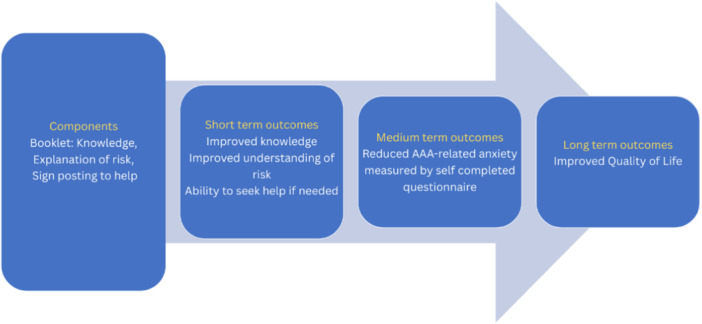
Programme theory for the information booklet.

Following workshop 1, we developed a simple logic model showing how the written information might improve patients' health‐related quality of life (see Figure 1). We identified a review of theories in patient health information seeking, but there were none that were fully relevant to the intervention [59]. One theory was identified as partially relevant: the Comprehensive Model of Information Seeking, which considers the influence of pre‐existing factors such as education and income, personal experience of an illness, perceived health threat, and personal beliefs about medical procedures and preventative behaviours on health information seeking [60]. This shaped our thinking by highlighting the following: health information can help psychological adaptation to a disease; provision of information as a leaflet or booklet can remove barriers to information‐seeking; information seeking is an iterative process; and information must be salient to individuals. This gave us confidence that an information booklet might help men in AAA surveillance if we ensured that the information was salient to them.

#### 3.2.4 Contribution of Workshop 2

In workshop 2, men and family members suggested changes to the booklet, and confirmed their requirements for how the intervention should be used. Specifically, they suggested that the booklet could be given more than once over the course of surveillance. Further refinements included the need for more information on possible symptoms to look out for.

### 3.3 Phase 3: Acceptability of the Intervention

#### 3.3.1 Response Rate

21% (31/150) of men in surveillance agreed to read the booklet and complete a telephone questionnaire, but of these, only 15% (23/150) completed the telephone questionnaire. Reasons for non‐completion after accepting the invitation included being too busy, on holiday, unwell, or in hospital. The response rate varied by IMD quintile (*p* < 0.001) with the two most socially deprived quintiles having the lowest completion rate of 8.5% (9/106) and the three most affluent quintiles having a completion rate of 32% (14/44).

#### 3.3.2 Characteristics of the Sample

Although the response rate was significantly lower for men from the most socially deprived areas, there was relatively good representation from them in the sample. Three men had received surgery to stabilise a large AAA. Only one of the 23 respondents (4%) was from an ethnic minority group.

#### 3.3.3 Helpfulness of the Booklet

100% of respondents said they would recommend the booklet. 87% (20/23) of respondents found the booklet very or quite helpful. They felt that it covered everything they would want to know about AAA. In open comments, eight respondents described how previous written information they received was not as detailed or comprehensive as the booklet. Suggestions for improvement included: bigger and clearer diagrams of the anatomy of an AAA, and alternatives to website links for further information, because 9/23 respondents stated that they would not or could not use them.

Knowledge needs appeared to be influenced by the size of AAA. Those with larger AAAs wanted more information about their treatment options. One respondent could not understand why surgery was not offered straight away, regardless of the size of the AAA, when patients were younger and fitter when first diagnosed.

#### 3.3.4 Timing and Frequency of the Booklet

A majority of respondents, 64% (14/23), thought the booklet should be given when first diagnosed with AAA. 27% (6/23) felt it would be useful to receive it in two parts, the first part when invited for screening, and the rest if diagnosed with AAA to avoid receiving too much information too soon. 64% (14/23) of respondents felt that they should receive the booklet once, and they would keep it to refer back to. 26% (6/23) of the respondents felt they should be given it again if their AAA changed size, that is, from small to medium or medium to large, or if there was new or updated information about AAA surveillance and management.

#### 3.3.5 Types of Information: Verbal Versus Written

In open comments, all respondents reported receiving good verbal information from the screening technicians and specialist nurses. Three respondents felt that the verbal information they received was enough, and two felt a booklet was needed to reinforce the verbal information they received.

#### 3.3.6 Potential Benefits: Increased Knowledge and Feeling Less Worried About AAA

83% (19/23) of the respondents agreed or strongly agreed that they knew more about AAA after reading the booklet. 65% (15/23) agreed or strongly agreed that they felt less worried about having AAA (Note that a validated scale to measure anxiety before and after reading the booklet was not used). None of the respondents reported that they felt more worried about AAA after reading the booklet.

#### 3.3.7 Satisfaction With the Booklet

The validated QQ‐10 showed that the booklet was acceptable to respondents, with an overall ‘value’ score of 91 out of a possible score of 100 (SD 16.1) and ‘burden’ score of 8 out of 100 (SD 10.6). There was no significant difference in ‘value’ and ‘burden’ scores based on IMD. Those men living in the most socially deprived areas scored the booklet slightly higher in terms of ‘value’ (score 97, SD 4.3) and slightly lower in terms of ‘burden’ (score 6, SD 8.1) than those living in more affluent areas (scores 87, SD 15.6 and 11, SD 12.8 respectively). Statistical comparison for this data set was not undertaken due to the small sample size.

#### 3.3.8 Public Awareness of AAA

In open comments, respondents described how they had had no idea what AAA was prior to being invited for screening, and that the existence and risk of an AAA diagnosis should have a higher public profile, similar to campaigns around prostate cancer, for example. They suggested posters in GP surgeries, and on billboards or buses, as well as TV adverts. They wanted this information to be more accessible, and not simply on the internet, because some of them did not like using computers or have access to them.

#### 3.3.9 Refining the Booklet

The booklet was refined again after this feedback, including shortening the title, removing or replacing some images and merging and reordering some sections. Telephone numbers were added where possible to information links because some men did not access information digitally.

### 3.4 Description of the Final Information Booklet

The final intervention was a 16‐page A5 booklet with 15 sections covering different aspects of AAA, including: ‘Why have I got AAA’, ‘Do's and Don'ts’ when diagnosed with AAA, ‘what will happen to my AAA over time’ and ‘who is involved in my care’. There were four photographs and four images. It included the risk of rupture for different sizes of AAA to allow some tailoring to individual men (see Appendix 5 for a copy of the final version of the intervention). It differed from existing AAA leaflets by showing the risk of dying in surveillance from AAA, describing how AAA is measured and why measurements might differ, communicating information in ways that have meaning to patients, for example, do's and don'ts, offering more detail on how to make lifestyle changes, and offering information about whether it is safe to lift things.

## 4 Discussion

### 4.1 Summary of Findings

An information booklet was developed with men with AAA, family members and service providers to help reduce uncertainties about AAA and AAA surveillance. Overall, the booklet was found to be acceptable to the men in AAA surveillance who reviewed it. They felt it covered AAA in more detail than other written information they may have already received. Despite most men in the sample reporting that they received good verbal information from the screening staff, they felt the booklet helped reinforce this and provided reassurance. The booklet was designed to be given to men when they are diagnosed with an AAA. There is evidence linking patient uncertainty with anxiety, and there is potential for this information booklet to help men in AAA surveillance to manage AAA‐related anxiety.

### 4.2 Context of Other Research

The need for more information is a common theme in AAA research and guidance in different countries. Recent research about men in surveillance with large AAAs identified the need for earlier provision of more detailed information and signposting to quality online information [61]. This was so they could be better prepared if they reached the stage where an intervention for their AAA was needed [61]. International guidance identifies the need for more information for patients in AAA surveillance to help them manage any AAA‐related anxiety [14]. In addition, the need for more accessible, understandable, comprehensive and consistent high‐quality information has been advocated for people in surveillance programmes for other conditions such as cancer [15, 17, 18]. Despite information leaflets already existing for AAA [62, 63, 64, 65], screening staff and men in AAA surveillance in the UK felt there was still an information gap, and that written information rather than psychological support would help alleviate uncertainties men may have about their AAA [12, 13]. The booklet co‐designed in this study could help address uncertainties by providing one comprehensive booklet that fulfils men's knowledge needs when they are first diagnosed with AAA.

More recent research has highlighted the relationship between information and anxiety in the context of studying awareness of the hereditary nature of AAA [66]. This study recommended better communication of risk as a way of alleviating anxiety about AAA [66]. This supports the potential for our information booklet to help men with AAA to manage anxiety because the risk of rupture for men in the national screening programme is documented within the booklet. Finally, researchers in Sweden have been developing and testing an intervention to manage anxiety further down the AAA pathway when patients are offered surgery [67]. Interestingly, this intervention is an electronic information‐based App. It could be argued that electronic and non‐electronic information sources for AAA are needed, as nearly half the men who reviewed our booklet expressed not having access to or lacking confidence in using digital technology.

### 4.3 Strengths and Limitations

There were two strengths to this research. First, we have addressed international guidance on information needs for men in AAA surveillance [14] by identifying the type of information needed through stakeholder engagement of service providers and patients. Second, we worked with patients and their family members, and clinicians on our research team, to co‐design an intervention so it was shaped by end‐users. There were six limitations. First, we failed to include men from ethnically diverse communities because only one man from South East Asian heritage was involved (he reviewed the booklet in the acceptability study). It could be argued that the lack of diversity in recruitment across the development and acceptability study could be expected given the lower prevalence and lower screening uptake for AAA in ethnic minority groups [68]. However, this remains a limitation because AAA affects men in all ethnic groups. Second, the response rate to the acceptability survey was low. It is possible that only men with information needs agreed to participate in the survey, and a higher response rate would reveal a lack of utility of the booklet. It is also the case that we would have obtained a higher response rate had we not tried to improve representation from men in socially deprived communities. Third, the use of the validated QQ‐10 tool to measure the face validity and utility of the booklet is a strength because it allows comparison with other studies. The high ‘value’ and low ‘burden’ score results from the QQ10. In this study were found to be comparable to other studies [69, 70, 71]. However, it has not been validated to assess information booklets. Fourth, the acceptability survey was based on a small unrepresentative sample. However, using a telephone administered rather than a postal survey allowed for detailed feedback to be gained, and the effort to include more men from socially deprived communities—who may need information more than other socio‐economic groups—went some way to counteract this limitation. Fifth, the literature review we undertook was not systematic and may have missed some helpful publications. Finally, this research was undertaken about men in a national screening programme. In the UK, men and women with AAA who are diagnosed outside the screening programme undergo surveillance in individual hospitals. The booklet has not been written for women, and the risk information within it would need to be adapted for women.

### 4.4 Implications

Several leaflets currently exist providing information about AAA at different stages of the surveillance process in the UK. Despite this, service providers and men felt that a lack of information about the disease and screening contributed to uncertainty about AAA. We developed a comprehensive booklet covering different aspects of AAA surveillance and management with the intention of it being made available to all men when they are first diagnosed with an AAA in the NHS AAA Screening Programme. We hope this will help men to manage any concerns or uncertainties their new diagnosis may cause.

Contact with, and verbal information received from, the screening technicians and the vascular nurse specialists was deemed highly valuable by participants throughout this body of research. All men entering or moving to 3 monthly surveillance in the UK NHS AAA Screening Programme are currently offered an appointment with a nurse specialist. As some men remain under surveillance for a number of years and are not routinely offered further appointments with a nurse specialist, beyond moving to 3 monthly surveillance, this highlights the need for written information to be supplied on a regular basis. The role of specialist nurses in supplying consistent and comprehensive verbal and written information in a timely manner to men in the NHS AAA Screening Programme surveillance is an important factor in addressing information needs and reducing uncertainty about AAA in the future. The booklet may be useful for men undergoing AAA surveillance outside any national screening programme in the UK and other countries but would need to be adapted for use by women because they have different rupture rates from men.

## 5 Conclusions

An information booklet was co‐designed to address uncertainties experienced by men with AAA in an AAA surveillance programme. The information booklet could be useful to other national and regional AAA screening programmes internationally that focus on men. Uncertainties may lead to anxiety, so future research should measure the impact of the information booklet on AAA‐related anxiety.

## Author Contributions

**Cheryl Grindell:** investigation, formal analysis, writing – original draft, writing – review and editing. **Jane Hughes:** investigation, formal analysis, writing – original draft, writing – review and editing. **Elizabeth Lumley:** conceptualisation, funding acquisition, methodology, supervision, writing – review and editing. **Alan Elstone:** conceptualisation, funding acquisition, methodology, writing – review and editing. **Jo Hall:** conceptualisation, funding acquisition, methodology, writing – review and editing. **Jonathan Michaels:** conceptualisation, funding acquisition, methodology, writing – review and editing. **Akhtar Nasim:** conceptualisation, funding acquisition, methodology, writing – review and editing. **Stephen Radley:** conceptualisation, funding acquisition, methodology, writing – review and editing. **Phil Shackley:** conceptualisation, funding acquisition, methodology, writing – review and editing. **Niall MacGregor Smith:** conceptualisation, funding acquisition, methodology, writing – review and editing. **Gerry Stansby:** conceptualisation, funding acquisition, methodology, writing – review and editing. **Emily wood:** conceptualisation, funding acquisition, methodology, writing – review and editing. **Alicia O'Cathain:** conceptualisation, funding acquisition, methodology, supervision, formal analysis, writing – review and editing.

## Ethics Statement

This study received ethics approval from the Wales REC6 Ethics Committee, IRAS project ID 321528.

## Consent

All participants provided written consent to participate.

## Conflicts of Interest

In the interests of transparency, the authors would like to highlight that three of the authors have roles in the NHS AAA Screening Programme; Alan Elstone is the Professional Clinical Advisor (Nursing), Mr Akhtar Nasim is the National Surgical Lead, and Professor Gerry Stansby is the Research Lead.

## Supporting information

Appendix 1_Searching for relevant literature.

Appendix 2_ The sampling approach.

Appendix 3_Men's View of the Booklet Questionnaire.

Appendix 4_Learning for the intervention from a literature search.

Appendix 5_Intervention Booklet 27.01.2026.

Appendix 6_What the developed booklet adds to existing leaflets.

## Data Availability

The data that support the findings of this study are available upon request from the corresponding author. The data are not publicly available due to privacy or ethical restrictions.
