# Supplementary material for: Developing and Assessing the Acceptability of an Information Booklet for Patients in Surveillance for Abdominal Aortic Aneurysms: An Intervention Development Study
Source: Health Expect. 2026 Mar 10;29(2):e70631. doi: 10.1111/hex.70631 (PMC12976147; doi:10.1111/hex.70631)
Supplement: Supplementary file 1 — Appendix 1_Searching for relevant literature. [file HEX-29-e70631-s003.docx]

**Appendix 1 Searching for relevant literature**

We were interested in information leaflets designed for patients in healthcare settings, particularly conditions similar to AAA where there is ongoing surveillance and where levels of risk might be of particular concern to patients. We asked our clinical team members for any relevant sources and carried out electronic database searching. We used ‘pearl growing’ (37) where particularly relevant or authoritative articles were used to search for further sources (38). We used some papers identified by our team as pearls and carried out electronic searches using PubMed. We also carried out an initial search using the term “information for patients” which retrieved a vast set of 443,000 citations published in the last ten years. We sorted these by best match and screened the first 20 pages (200 citations) at which point the relevance of the citations became limited. We applied the review or systematic review filter to the search, which yielded over 65000 citations, and again sorted by best match and screened the first 200. We carried out a second search using the term “information on AAA” and retrieved and screened 475 citations. In addition to this database searching, we scrutinised the reference list of relevant articles and used the “cited by” and “similar to” features to seek further potentially relevant articles.
