# Supplementary material for: Developing and Assessing the Acceptability of an Information Booklet for Patients in Surveillance for Abdominal Aortic Aneurysms: An Intervention Development Study
Source: Health Expect. 2026 Mar 10;29(2):e70631. doi: 10.1111/hex.70631 (PMC12976147; doi:10.1111/hex.70631)
Supplement: Supplementary file 2 — Appendix 2_ The sampling approach. [file HEX-29-e70631-s006.docx]

**Appendix 2 The sampling approach**

We sent an invitation to 50 men who had responded to the patient survey we had previously undertaken.

Participants’ postcodes were used to calculate the Indices of Multiple Deprivation (IMD) and convert these to quintiles (28). We were then able to select men from all five quintiles.

10/50 completed the survey, with lower representation from men in the socially deprived quintiles. Given that men in these quintiles were more likely to report AAA-related anxiety (9) we changed our approach to sampling to include more representation from men in deprived quintiles.

In the second sample, 20 men were selected from socially deprived areas only. Of these, only 2/20 completed the survey.

We then selected a further sample and sent a further 61 invites to men living in socially deprived areas. From this sample, only 3/61 completed the survey. Finally, we selected a further 19 men from all quintiles where 8/19 completed the survey.
