# Supplementary material for: Developing and Assessing the Acceptability of an Information Booklet for Patients in Surveillance for Abdominal Aortic Aneurysms: An Intervention Development Study
Source: Health Expect. 2026 Mar 10;29(2):e70631. doi: 10.1111/hex.70631 (PMC12976147; doi:10.1111/hex.70631)
Supplement: Supplementary file 3 — Appendix 3_Men's View of the Booklet Questionnaire. [file HEX-29-e70631-s005.pdf]

Participant ID:

Men's Views of the Booklet

Thank you for reading the booklet and offering to give your views about it. We do not mind if you have found problems with it. We are looking for honest views, so we know what to do next.

1. How helpful did you find the booklet?

Very helpful................................ ☐
Quite helpful............................... ☐
Neither helpful or not helpful...... ☐
Not very helpful.......................... ☐
Not at all helpful......................... ☐

Why? Any improvements we can make?

Participant ID:

2.Going through the different sections of the booklet from section 1 to section 13:
Were any sections particularly helpful? Why?
Were any unhelpful? Why?

	**Helpful**<br>**Why?**	**Unhelpful**<br>**Why?**
**Section 1. What is AAA?**		
**Section 2. Screening for AAA**		
**Section 3. Why have I got AAA?**		
**Section 4. What are the different sizes of AAA?**		
**Section 5. Do's and Don'ts**<br>**How can I reduce the chance of my AA growing or bursting?**		

**What am I allowed to do when I have AAA?**		
**Section 6. Are there symptoms I should look out for?**		
**Section 7. Benefits of having regular scans**		
**Section 8. Why is AAA not treated immediately?**		
**Section 9. How many times a year do I need a scan?**		

**Section 10. How is AAA measured?**		
**Section 11. What will happen to an AAA over time?**		
**Section 12. Risks of AAA bursting**		
**Section 13. What happens if an AAA becomes large?**		

**Section 14. Who is involved with my care?**		
**Section 15. My Questions**		
**More information**		

***3.Please circle the answers below each of the following 10 statements that best fit your feelings about the booklet**

The information leaflet is relevant to AAA screening

Strongly agree	Mostly agree	Neither agree nor disagree	Mostly disagree	Strongly disagree
☐	☐	☐	☐	☐

The information leaflet is easy to understand

Strongly agree	Mostly agree	Neither agree nor disagree	Mostly disagree	Strongly disagree
☐	☐	☐	☐	☐

Participant ID:

The information leaflet includes all important aspects of AAA screening that concern me

Strongly agree	Mostly agree	Neither agree nor disagree	Mostly disagree	Strongly disagree
☐	☐	☐	☐	☐

The information leaflet will help communicate/people understand about AAA screening

Strongly agree	Mostly agree	Neither agree nor disagree	Mostly disagree	Strongly disagree
☐	☐	☐	☐	☐

I enjoyed reading the information leaflet

Strongly agree	Mostly agree	Neither agree nor disagree	Mostly disagree	Strongly disagree
☐	☐	☐	☐	☐

I would be happy to use the information leaflet as part of AAA screening

Strongly agree	Mostly agree	Neither agree nor disagree	Mostly disagree	Strongly disagree
☐	☐	☐	☐	☐

The information leaflet is too long

Strongly agree	Mostly agree	Neither agree nor disagree	Mostly disagree	Strongly disagree
☐	☐	☐	☐	☐

The information leaflet is not needed

Strongly agree	Mostly agree	Neither agree nor disagree	Mostly disagree	Strongly disagree
☐	☐	☐	☐	☐

The information leaflet is too complicated

Strongly agree	Mostly agree	Neither agree nor disagree	Mostly disagree	Strongly disagree
☐	☐	☐	☐	☐

The information leaflet upset me

Strongly agree	Mostly agree	Neither agree nor disagree	Mostly disagree	Strongly disagree
☐	☐	☐	☐	☐

4.How did you feel after reading the booklet?

I felt like I knew more about AAA

Strongly agree	Mostly agree	Neither agree nor disagree	Mostly disagree	Strongly disagree
☐	☐	☐	☐	☐

I felt less worried about having AAA

Strongly agree	Mostly agree	Neither agree nor disagree	Mostly disagree	Strongly disagree
☐	☐	☐	☐	☐

I felt more worried about having AAA

Strongly agree	Mostly agree	Neither agree nor disagree	Mostly disagree	Strongly disagree
☐	☐	☐	☐	☐

5. Would you recommend we give this booklet to men in surveillance?

If so, when and how often?

If not, why?

6. How often would you use it? One off or return to it over time?

Were any important issues, problems or concerns missed out?

Do you have any additional comments or suggestions to make about the booklet?

Do you have any comments or suggestions about the AAA screening programme as a whole?

THANK YOU

*** QQ-10©**
