# Supplementary material for: Developing and Assessing the Acceptability of an Information Booklet for Patients in Surveillance for Abdominal Aortic Aneurysms: An Intervention Development Study
Source: Health Expect. 2026 Mar 10;29(2):e70631. doi: 10.1111/hex.70631 (PMC12976147; doi:10.1111/hex.70631)
Supplement: Supplementary file 4 — Appendix 4_Learning for the intervention from a literature search. [file HEX-29-e70631-s004.docx]

**Appendix 4 Learning for the intervention from a literature search**

| Contribution | Learning for intervention | Reference |
| --- | --- | --- |
| Effect of information | Giving a patient information leaflet improved knowledge and satisfaction  Self-management education in cancer improved anxiety but it was not possible to say which components/aspects created change | (51)  (36) |
| Changing behaviour e.g. addressing risk factors | Video-based education, promoting gains not losses, are effective in changing behaviour but not addictive behaviour | (54) |
| Quality of AAA information | Lots of websites offering information about AAA repair but they are of poor quality.  Healthcare providers should focus on the provision of better AAA-focused patient information (e.g., appropriately referenced, regularly reviewed, and limiting advertisements where possible) because information is weak and has a high reading age.  Youtube videos for myocardial infarction were of high quality but those for AAA diagnosis and treatment were poor quality in terms of completeness and reliability | (32)  (50)  (35)  (44) |
| Creating information | If creating a web-based intervention, follow the steps of co-design, assess it and then refine it  Patient involvement may improve success | (48)  (45) |
| Measuring quality of information | MEQIP is a tool for measuring quality of decision aids. It may be worth considering when developing our intervention (Modified Ensuring Quality Information for Patients Tool). 36 item checklist <http://refhub.elsevier.com/S1078-5884(24)00015-7/sref15>  GQS Global Quality Scores measures quality of websites and DISCERN measures reliability | (32)  (35) |
| Examples of high quality information | Six high quality websites identified that may offer source of well written information relevant to our booklet | (32) |
| Communicating information | Natural frequencies are best  Using pictures and icons (cartoons, photos, pictograms) improves understanding, especially in low health literacy population if they only have a few words in the picture.  Tabular and icon fact boxes are equally effective.  Summarising highlights aids communication  Question Prompt Lists for patients may change the topics discussed in a consultation but show limited impact on psychological outcomes | (40,53)  (41,47,53)  (39)  (40,41)  (37) |
| Information on risk | Risk is often left out of information when it should be there | (32) |
| Communicating risk | Visual aids and absolute risk formats can improve patients' understanding of probabilistic information, whereas numbers needed to treat can lessen their understanding. Visual aids are helpful.  Patients want personalised information but need to be supported when receiving it so they can understand it  Can include comparative data with people’s personalised risk e.g. better than average  Doctors use persuasive language to steer patients to their preferred treatment  Verbal descriptors including “common”, “uncommon” and “rare” lead to an overestimation of the probability of adverse effects compared to numerical information. Numbers result in more accurate estimates and increase satisfaction and likelihood of medication use. | (40,58)  (55)  (55,56)  (57)  (34,42) |
