# Supplementary material for: Developing and Assessing the Acceptability of an Information Booklet for Patients in Surveillance for Abdominal Aortic Aneurysms: An Intervention Development Study
Source: Health Expect. 2026 Mar 10;29(2):e70631. doi: 10.1111/hex.70631 (PMC12976147; doi:10.1111/hex.70631)
Supplement: Supplementary file 5 — Appendix 5_Intervention Booklet 27.01.2026. [file HEX-29-e70631-s002.pdf]

Abdominal Aortic Aneurysms

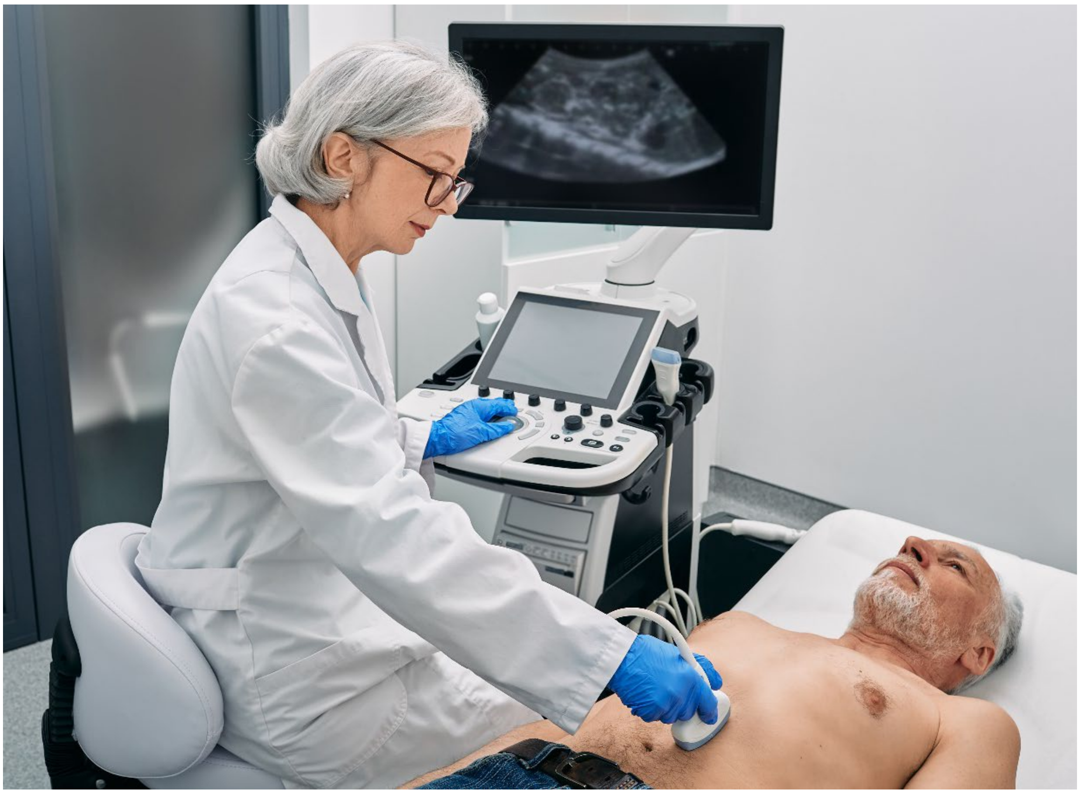

This booklet is aimed at people who have an Abdominal Aortic Aneurysm and have regular scans

Table of contents

1.What is an Abdominal Aortic Aneurysm (AAA)?............. 2
2.Screening for Abdominal Aortic Aneurysm 2
3.Why have I got an Abdominal Aortic Aneurysm? 3
4.What are the different sizes of AAA? 3
5. How can I reduce the chance of my AAA growing or bursting? 4
6.Are there symptoms I should look out for?.................... 7
7.Benefits of having regular scans (surveillance) 8
8.How many times a year do I need a scan? 9
9.How is an AAA measured?... 9
10.What will happen to an AAA over time? 9
11.Risks of AAA bursting 11
12.What happens if an AAA becomes large?................... 12
13.Who is involved with my care? 12
14.My Questions... 14

Version last updated 27/01/2026

1.What is an Abdominal Aortic Aneurysm (AAA)?

The aorta is the main blood vessel that supplies blood to your body. It runs from your heart down through your chest and abdomen (tummy).

In some people, as they get older, the wall of the aorta in the abdomen can become weak. It can then start to bulge. This bulge is called an aneurysm.

Abdominal Aortic Aneurysm is usually called AAA or triple A for short.

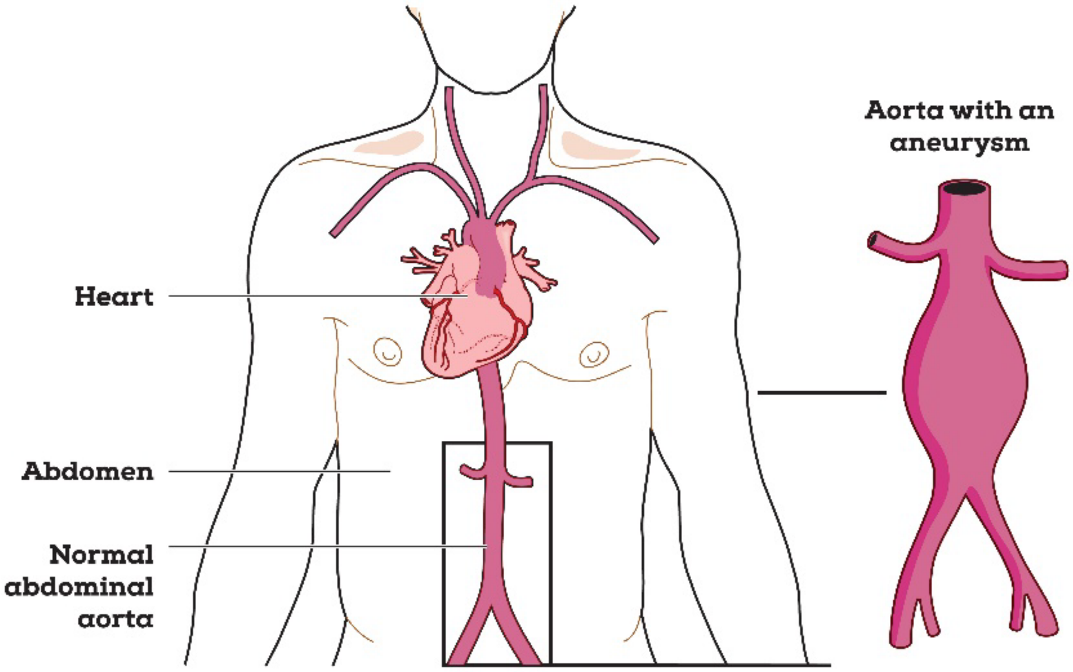

2.Screening for Abdominal Aortic Aneurysm

AAA screening is recommended for anyone assigned male at birth. This includes men, trans women and non-binary people. AAA screening is offered to males over the age of 64. Males are 4 times more likely to have AAA than females, which is why females are not offered screening. Around 300,000 males are screened each year in England 2000 are found to have an AAA. AAA is sometimes found in people when they have

investigations for health problems. These people go into surveillance too.

3.Why have I got an Abdominal Aortic Aneurysm?

The chance of having an AAA can increase if:

- you smoke
- you have high blood pressure, diabetes or chronic obstructive pulmonary disease (COPD)
- people in your family have had AAA
- you are white
- you are older

It is part of a wider problem with arteries which happens as people get older. This is called arterial disease. Even people who look after themselves by having a healthy lifestyle can develop problems with their arteries. Having cancer or other conditions does not cause AAA.

4.What are the different sizes of AAA?

Usual size of an aorta

The usual size of an aorta is less than 3cm across. If an aorta is more than 3cm across, males are invited to have regular scans (surveillance).

Small aneurysm

If your aorta is between 3 cm and 4.4 cm wide, you have a small aneurysm.

Medium aneurysm

If your aorta is between 4.5 cm and 5.4 cm wide, you have a medium aneurysm.

Large aneurysm

If your aorta grows to 5.5 cm wide, you have a large aneurysm. Men with a large aneurysm are offered an appointment with a specialist team for more tests and possible treatment e.g. surgical repair.

5. How can I reduce the chance of my AAA growing or bursting?

You can:

- **Stop smoking (if you smoke).** You are three times more likely to quit smoking with support. You can contact your local stop smoking service or speak to your GP or someone at the AAA screening service. Stopping smoking is the best thing to do to slow the growth of AAA. This will also significantly reduce your risks of stroke, heart attack and cancer. The effect of vaping is not known.
- **Manage your blood pressure.** It is less likely your aneurysm will grow quickly or burst if your blood pressure is normal. Speak to your GP or practice nurse to get your blood pressure checked. The GP may suggest taking medicines to manage your blood pressure. They may also suggest other medicines, such as statins or a blood thinner, to lower your risk of having a heart attack or stroke.

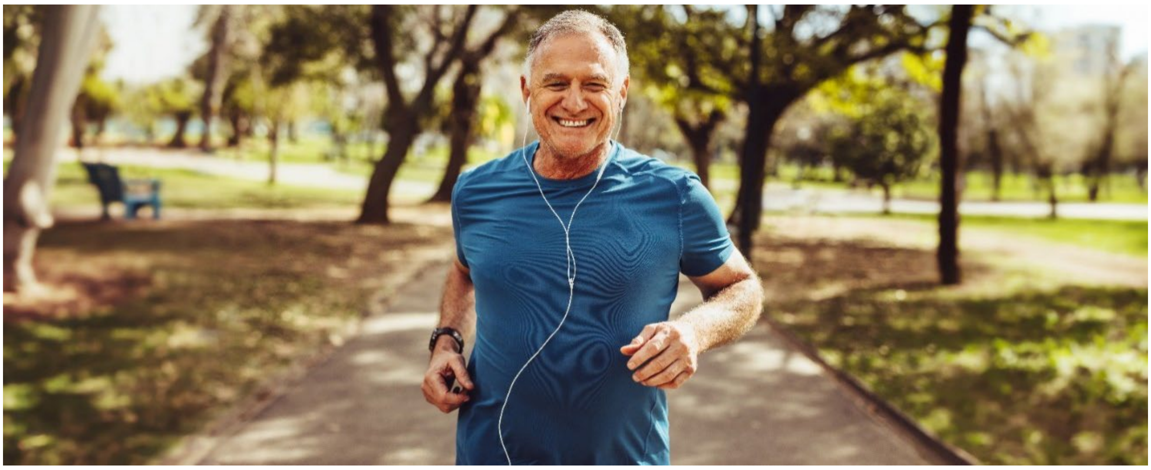

- **Keep active.** Physical activity is not just about exercise classes and working up a sweat in the gym. It is everyday activities such as housework, walking to work, gardening and dancing to the radio in your kitchen. Please follow this link for recommendations for exercise:https://www.who.int/news-room/fact-sheets/detail/physical-activity
- **Maintain a healthy weight or lose weight if you need to.** If you need surgery, any risk from having surgery is lower if you have a healthy weight. You do not have to see a GP about this but if you are concerned about your health, then you may need to talk to a GP to ensure you do things safely.
- **Drink sensibly.** People are advised not to drink more than 14 units a week on a regular basis. Spread your drinking over 3 or more days if you regularly drink as much as 14 units a week. If you want to cut down, try to have several drink-free days each week. 14 units is equivalent to 6 pints of average-strength beer or 10 small glasses of lower-strength wine: https://www.nhs.uk/live-well/alcohol-advice/calculating-alcohol-units/

Make an appointment to talk to the vascular nurse specialist at your screening unit. They can give you advice about how to improve your health. Doing these things will also make it less likely you will have a heart attack or stroke because they will improve your arterial disease.
It is also important to manage your cholesterol/lipid levels to maintain your arterial health and reduce the risk of stroke and heart disease. This is usually done with the use of statin medication from your GP. It is recommended that males with AAA take an anti-platelet medication such as Aspirin or Clopidogrel daily as well as medication to control blood pressure. The main purpose of statins and Aspirin is to reduce future risk of cardiovascular events such as heart attack or stroke.

What am I allowed to do when I have AAA?

✓ **Exercise**

It is safe to walk and cycle and do general tasks such as lifting shopping bags etc. If you are considering heavy lifting or contact sports, get advice from the specialist nurse at your screening unit.

✓ **Driving**

- If you have a car or motorcycle licence you can drive if your AAA is smaller than 6.4 cm. You must tell the DVLA if your AAA is 6 cm or more.
- You are not allowed to drive if your AAA is 6.5 cm or more. If it is, you must give up your licence. You will get your licence back if your AAA is successfully treated.
- If you are a bus, coach or lorry driver you must tell the DVLA whatever the size of your AAA. You are not allowed to drive if it is

5.5 cm or more and you must give up your licence. You will get your licence back if your AAA is successfully treated.

- Here is the link and phone number to the DVLA website: https://www.gov.uk/aneurysm-and-driving

  DVLA Medical Enquiries

  Telephone: 0300 790 6806

✓ **Air Travel**

- There is no increased risk if you travel by plane when you have an AAA. It is no more likely to burst because of cabin pressure at a high altitude than on the ground.
- You should declare your AAA when applying for travel insurance. You may be charged more or have AAA excluded from cover. It may be worth shopping around as some companies specialise in providing insurance for people with pre-existing medical conditions.

✓ **Having sex**

- You can continue to have sex.

6.Are there symptoms I should look out for?

In most cases you will not have any symptoms. An ultrasound test is usually the only way to find out if you have an AAA. But if an AAA becomes large it can sometimes cause a pulsating feeling in your abdomen, or persistent back pain.

If an AAA ruptures it will cause internal bleeding. This is a medical emergency, and you should call 999 immediately if you or someone with you has any of these symptoms:

- a sudden severe pain in the abdomen, back or lower back area
- feeling cold, clammy, sweaty, faint and breathless
- loss of consciousness

Follow this link for further information from the British Heart Foundation: https://shorturl.at/fJJr6

7.Benefits of having regular scans (surveillance)

If you have a small or medium AAA you will not need surgery at this stage. However, it is important to monitor the size of the AAA as you might need surgery if it gets bigger. Most AAAs get bigger very slowly, so many men with a small or medium aneurysm will never need surgery.

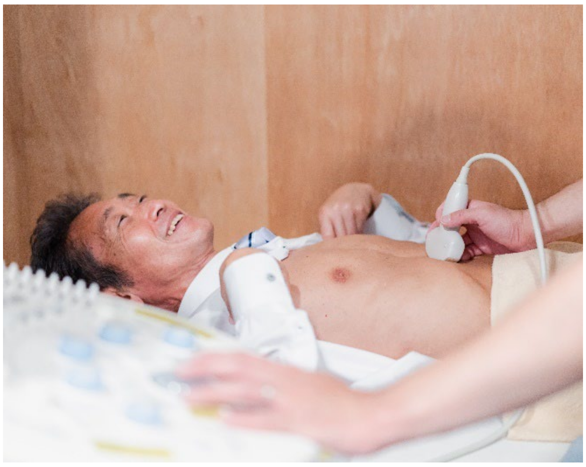

The easiest way to find out if your AAA is growing is to go for your regular scans.

Most AAAs do not burst. Surgery can be risky. It is riskier to have surgery for a small or medium AAA than waiting to have surgery. It is therefore considered safer for people to remain in surveillance until their AAA reaches 5.5cm.

8.How many times a year do I need a scan?

If you have a small sized AAA you will have a scan every **12 months**.
If you have a medium sized AAA you will have a scan every **3 months**.

9.How is an AAA measured?

An ultrasound scan is used, similar to that offered to pregnant women. The scan shows a picture of the aorta on a screen and a technician measures it. The aorta is measured from inside the front wall to the inside of the back wall, and also from side wall to side wall, of the widest part of the aorta.

Screening staff measure the AAA the right way, using ultrasound. Some people have other health problems and might have another type of scan such as a CT scan for that problem. If an AAA is measured using a different type of scan, the measurement may be different. The ultrasound measurement is the best way to measure an AAA.

You can ask the technician to show you your AAA next time you go for a scan. Some people have found it helpful to see their AAA.

10.What will happen to an AAA over time?

Do AAAs always grow?
No. Some AAAs do not grow.
Can growth slow down or stop?
Yes. Some AAA can stop growing.

Does growth increase as people get older?

AAA's do not specifically grow as people get older but their size may increase with time.

Can AAAs shrink?

No. The heart pumps blood at pressure through the aorta so this keeps it expanded.

Can anything I do stop the AAA growing or slow it down?

Yes. See Section 5 on page 4. There is no guarantee this will stop growth, but it could help to slow it down. Taking these actions can also reduce your risk of heart disease, stroke and arterial disease.

Does anything trigger growth?

The main risk for growth is smoking.

Does anything trigger the AAA bursting?

In most cases there is no obvious cause of an AAA bursting. It is important to remember that AAAs do not burst in most people who have them and that bursting is rare if the AAA is less than 5.5 cm. (People with high blood pressure are more likely to have an AAA that bursts and smoking increases risk of AAA growth and rupture).

What are the chances of an AAA bursting when in surveillance (having regular scans)?

It is important to remember that AAAs do not burst in most people who have them.

There is a very low chance that an AAA could burst when in surveillance. The screening service keeps a close eye on AAAs so that the chance of there being a problem is very low.

Different sizes of AAA have different risks, and this is why men with medium AAAs are scanned more often.

11.Risks of AAA bursting

For an AAA measuring between 3 to 4.4 cm (small AAA)

About 3 males in 10,000 in AAA surveillance will have a burst AAA before their next annual screen. This is a very low risk. The picture shows the risk for 100 males.

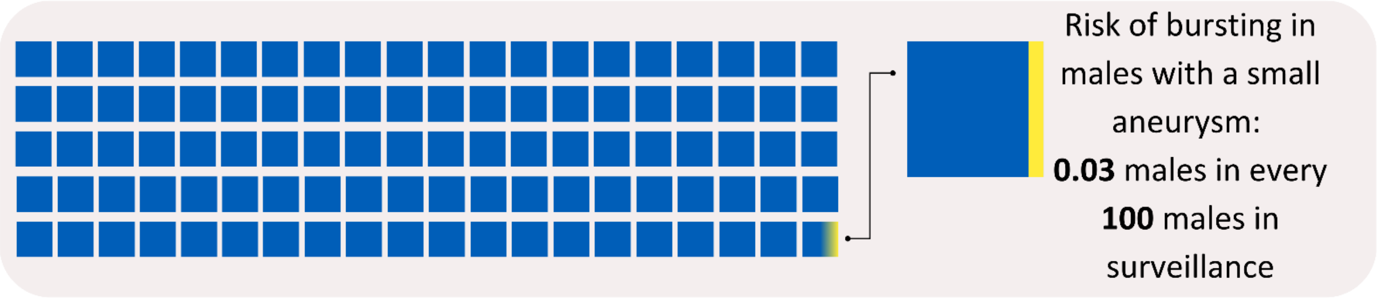

For an AAA measuring between 4.5 to 5 cm (medium AAA)

About 3 males in 1000 in AAA surveillance will have a burst AAA before their next screen. This is a very low risk. The picture shows the risk for 100 males.

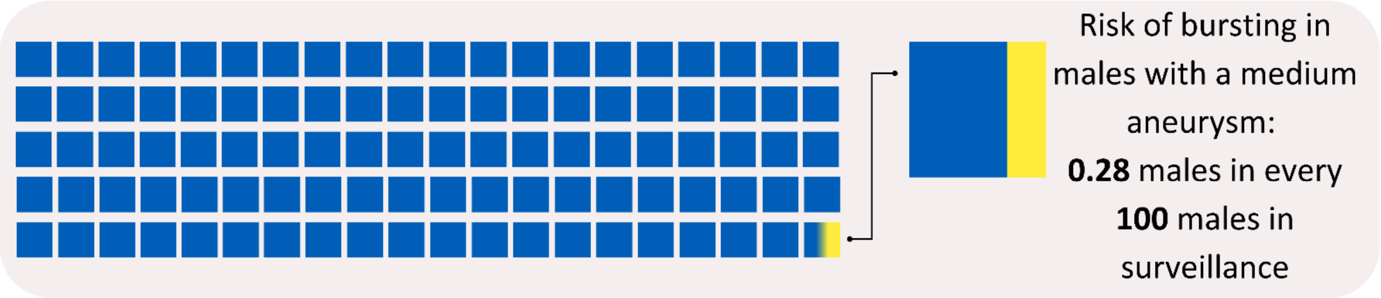

For an AAA measuring between 5cm and 5.4cm

About 4 males in 1000 in AAA surveillance will have a burst AAA before their next screen. This is a very low risk. The picture shows the risk for 100 males.

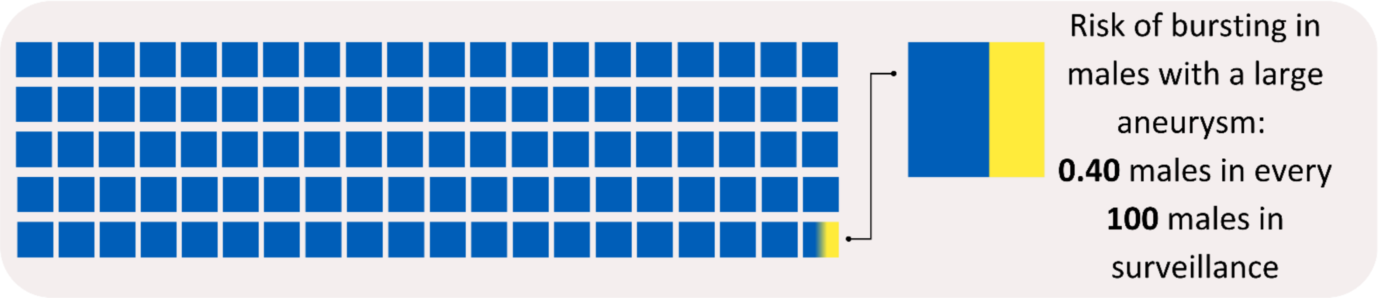

This is the risk per year.

12. What happens if an AAA becomes large?

If an AAA becomes large (5.5cm), the screening service will refer the patient to a specialist team in a hospital with a vascular centre. They will carry out some more tests. The specialist team will discuss the potential options available with the patient and their family. Patients will be involved in the decision-making process about how the AAA is managed going forward.

During the time when a large AAA is not treated, a patient may need to stop driving and to check health-related insurance, such as travel insurance.

More information is available here about options and how to make decisions about them:
https://www.england.nhs.uk/wp-content/uploads/2024/09/PRN00675-v-making-a-decision-about-abdominal-aortic-aneurysm-AAA.pdf

13. Who is involved with my care?

Screening Technicians

- Screening technicians explain the scan, gain consent, perform the scan, and give the results. You can ask them about the scan. You can ask to see your AAA. You will see these screening technicians once a year or every three months.

Nurse Specialists

- Nurse Specialists provide physical and emotional support following a diagnosis of an AAA. You will be offered an appointment with a Nurse Specialist when you start having regular scans (on entering

surveillance). You will also be offered an appointment if you move from having scans once a year to having them every three months. They can discuss most aspects of AAA care and how to reduce your risk of AAA growth and cardiovascular disease.

Vascular Surgeons

- Vascular Surgeons discuss the possibility of surgery if your AAA reaches 5.5cm. They will decide with you whether surgery is in your best interests and the way forward with your AAA management. You will usually see them when your AAA reaches 5.5cm.

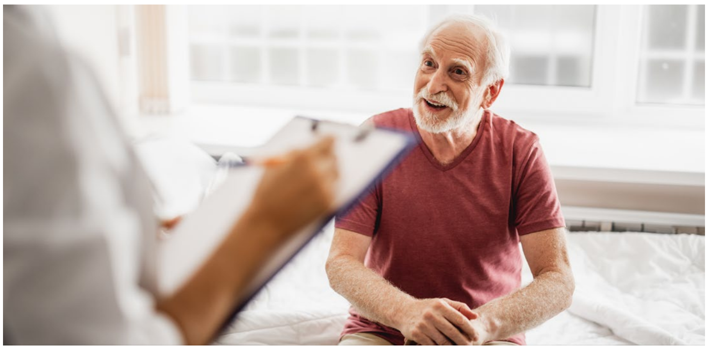

14.My Questions

Write any questions you have here so you remember to ask them next time you see a Nurse Specialist.

Places that have information about AAA

There are lots of places with reliable information. They are listed below.

NHS:

https://www.nhs.uk/conditions/abdominal-aortic-aneurysm/

Tel: 111

British Heart Foundation:

https://www.bhf.org.uk/informationsupport/conditions/abdominal-aortic-aneurysm

Tel: 0300 330 3322

Version last updated 27/01/2026

Circulation Foundation:
https://www.circulationfoundation.org.uk/patient_info/abdominal-aortic-aneurysm/

Tel : 020 7205 7151

DVLA (for information about driving with an AAA):
https://www.gov.uk/aneurysm-and-driving

Tel: 0300 790 6806

The diagrams on page 11 are based on this research paper. It is freely available on the internet.

Oliver-Williams C, Sweeting MJ, Jacomelli J et al (2019) Safety of men with small and medium abdominal aortic aneurysms under surveillance in the NAAASP. Circulation 139: 1371 to 1380.
It is freely available on the internet here.
https://www.ahajournals.org/doi/10.1161/CIRCULATIONAHA.118.036966

This booklet was put together by a research team funded by the National Institute for Health and Care Research in 2025. We asked patients and their families what information they wanted and worked with them to develop this booklet. We brought together information from the NHS website, NHS leaflets, and NHS decision aids offered to males in the AAA screening programme, and from clinical expertise in the research team (Nurse Specialist, Vascular Surgeons).

Version last updated 27/01/2026
