# Supplementary material for: Developing and Assessing the Acceptability of an Information Booklet for Patients in Surveillance for Abdominal Aortic Aneurysms: An Intervention Development Study
Source: Health Expect. 2026 Mar 10;29(2):e70631. doi: 10.1111/hex.70631 (PMC12976147; doi:10.1111/hex.70631)
Supplement: Supplementary file 6 — Appendix 6_What the developed booklet adds to existing leaflets. [file HEX-29-e70631-s001.docx]

**Appendix 6 What the developed booklet adds to existing leaflets**

|  | **NHS AAA screening leaflet – deciding whether to attend** | **NHS AAA screening booklet (updated July 2024)** | **NHS small AAA leaflet (online)** | **NHS medium AAA leaflet (online)** | **NHS large AAA decision aid (online)** | **What the PCAAS booklet adds** |
| --- | --- | --- | --- | --- | --- | --- |
| **The PCAAAS booklet contents** |  |  |  |  |  |  |
| What is a AAA | Yes | Yes | Just diagram | Just diagram | Yes | Nil |
| Screening for AAA | Yes | Yes | No | No | NA | Number of men screened each year. Coincidental finding with invests for other health problems |
| Why have I got an AAA | Yes | Yes | No | No | Yes | Information about arterial disease. Other conditions do not cause AAA eg. cancer |
| What are the different sizes of AAA | Yes | Yes | Yes | Yes | NA | Nil |
| Do’s and Don’ts  How can I reduce my AAA growing or bursting  What am I allowed to do | No  No | No  No | Framed as general health/generally keeping healthy  Yes driving and holiday insurance  General re: sports and hobbies | Framed as general health/generally keeping healthy  Yes driving and holiday insurance  General re: sports and hobbies | Framed specifically for AAA  Directs to GP for smoking cessation, managing BP , lifestyle changes and meds  Yes driving ,lifting, air travel | Framed specifically for AAA. Directs to nurse specialist for advice.  Info re: arterial health and risk of stroke and heart disease.  Includes specific examples eg. Lifting, walking, cycling  and sex |
| Are there symptoms I should look out for | No | Usually no symptoms | Signs and symptoms of rupture | Signs and symptoms of rupture | Pulsating feeling in abdomen | Nil but all info together in one place |
| Benefits of regular scans | Yes | Yes | No | No | Decision on scans dependent on treatment decision | Nil |
| Why is AAA not treated immediately | No | No | Yes | Yes | N/A | Nil |
| How many times a year do I need a scan | Yes | Yes | Yes | Yes | N/A | Nil |
| How is AAA measured | Yes | Yes | No | No | NA | Detail about how AAA is measured and other types of scans if you have other health problems |
| What will happen to a AAA over time | No | No | No | No | General re growth over time | Specific and new |
| Risk of AAA bursting | 10 year risk | No | Generic | Generic | Small AAA only  General increased risk if large AAA or over time | Risk of small medium and large given all in one place |
| What happens if AAAA becomes large | Yes | Yes | Yes | Yes | Yes | Nil |
| Who is involved in my care | No | No | No | No | No | All new |
| My Questions | No | No | No | No | No | All new |
| Where can I go for more info | Yes | Yes | Yes | Yes | Yes | Nil |
